# Health utilities and costs for neuromyelitis optica spectrum disorder

**DOI:** 10.1186/s13023-022-02310-z

**Published:** 2022-04-07

**Authors:** Dyfrig A. Hughes, Siobhan Bourke, Angela Jones, Rikesh Bhatt, Saif Huda, Kerry Mutch, Anu Jacob

**Affiliations:** 1 Centre for Health Economics and Medicines Evaluation, Ardudwy, Bangor University, Holyhead Road, Bangor, LL57 2PZ Wales UK; 2 Department of Health Services Research and Policy, The Australian National University, Canberra, Australia; 3 Department of Applied Health Research, University College London, London, UK; 4 The Walton Centre, Liverpool, UK; 5 The Cleveland Clinic Abu Dhabi, Abu Dhabi, United Arab Emirates

**Keywords:** Neuromyelitis optica spectrum disorder, Carers, Cost of illness, EQ-5D, Utility

## Abstract

**Background:**

Neuromyelitis optica spectrum disorder (NMOSD) is a rare, neurological disease that places a significant burden on patients, their carers, and healthcare systems.

**Objectives:**

To estimate patient and carer health utilities and costs of NMOSD within the UK setting.

**Methods:**

Patients with NMOSD and their carers, recruited via a regional specialist treatment centre, completed a postal questionnaire that included a resource use measure, the EuroQoL (EQ)-5D-5L, EQ-5D-VAS, Vision and Quality of Life Index (VisQoL), Carer Experience Survey (CES) and the Expanded Disability Status Scale (EDSS). The questionnaire asked about respondents’ use of health and community care services, non-medical costs, informal care and work capacity. Data were analysed descriptively. Uncertainties in costs and utilities were assessed using bootstrap analysis.

**Results:**

117 patients and 74 informal carers responded to the survey. Patients’ mean EQ-5D-5L and VisQoL health utilities (95% central range) were 0.54 (− 0.29, 1.00) and 0.79 (0.11, 0.99), respectively. EQ-5D-5L utility decreased with increasing EDSS score bandings, from 0.80 (0.75, 0.85) for EDSS ≤ 4.0, to 0.20 (− 0.29, 0.56) for EDSS 8.0 to 9.5. Mean, 3-month total costs were £5623 (£2096, £12,156), but ranged from £562 (£381, £812) to £32,717 (£2888, £98,568) for these EDSS bandings. Carer-reported EQ-5D-5L utility and CES index scores were 0.85 (0.82, 0.89) and 57.67 (52.69, 62.66). Mean, 3-month costs of informal care were £13,150 to £24,560.

**Conclusions:**

NMOSD has significant impacts on health utilities and NHS and carer costs. These data can be used as inputs to cost-effectiveness analyses of new medicines for NMOSD.

## Introduction

Neuromyelitis optica spectrum disorder (NMOSD) is a rare (1–2 people per 100,000) neurological, autoimmune disease typically characterised by episodes of optic neuritis, transverse myelitis, together with one or more other diagnostic criteria including the presence of serum aquaporin-4 antibodies [1]. Patients experience optic neuritis as pain which is rapidly followed by loss of acuity. Individuals affected by myelitis typically experience pain in the spine or limbs, mild to severe paralysis of the lower limbs, and loss of bowel and bladder control. Recurrent relapses of optic neuritis and/or myelitis, from which recovery is often incomplete, results in residual and accumulating impairment (such as blindness and paraplegia).

Conventionally managed with corticosteroids, azathioprine, mycophenolate mofetil and rituximab, new immunosuppressive treatments—including eculizumab, satralizumab, and inebilizumab—are changing the therapeutic landscape for NMOSD [2]. These treatments have different targets within the immune pathogenic process and while they are not curative, they reduce relapse rate and neurological deficit. However, they are very expensive. The annual cost of eculizumab is approximately £327,600 in the UK, based on four 300 mg vials every 2 weeks and a National Health Service (NHS) indicative price of £3150 per vial [3]. The costs of satralizumab and inebilizumab in the USA are $219,231 and $393,000 for the first year, respectively, and $190,000 and $262,000 per year thereafter [2].

In the UK, treatments for NMOSD are commissioned via NHS specialised services; and consequently, they compete with other specialised services for funding, and must therefore demonstrate value for money to gain routine adoption. Economic evaluations assess value for money by estimating the incremental cost associated with achieving additional quality-adjusted life years (QALYs). Within the technology appraisal programme of the National Institute for Health and Care Excellence (NICE), a cost per QALY below £20,000 to £30,000 is deemed to be cost-effective [4]. However, for Highly Specialised Technologies, the threshold increases to £100,000 (and exceptionally, up to £300,000) per QALY [4].

Highly effective treatments that prevent hospital admissions, reduce caregiver costs and improve health-related quality of life may conceivably achieve cost-effectiveness, even at these high prices. However, there is very limited evidence on the direct and indirect costs of care for patients with NMOSD, and considerable uncertainty surrounding the cost-effectiveness of treatments. NICE was unable to make a recommendation on eculizumab as the sponsor did not provide an evidence submission [5].

Improved accuracy and precision in the estimates of costs and health outcomes will result in more reliable inputs to economic models concerning treatments of NMOSD. This should provide decision makers greater confidence in the results of cost-effectiveness analyses. The aim of this research, therefore, was to estimate the costs associated with NMOSD, and measure health-related quality of life weights, expressed in terms of utilities, that would allow for the calculation of QALYs, given that a QALY is the time integral of utility.

## Methods

A sample of patients with NMOSD and their carers were recruited and consented to complete a postal survey which included a resource use questionnaire, the EuroQol (EQ)-5D-5L and visual analogue scale (VAS), the Carer Experience Scale, the Vision and Quality of Life Index (VisQoL) and the Expanded Disability Status Scale (EDSS) measures. The survey was undertaken between January 2016 and July 2018, following ethical approval that was granted by the London—Hampstead NHS Research Ethics Committee (reference 15/LO/1433).

### Patient questionnaire

Patient questionnaires were in three parts: (1) demographics (age and sex); (2) resources used or lost; and (3) health outcomes, in terms of health-related quality of life, health utilities and disease severity. Clinical characteristics were obtained from patients’ medical records, and included the duration since onset of NMOSD symptoms, length of time for referral to the treatment centre, and whether and how many relapses were experienced in the past year.

#### Resource use

The Database of Instruments for Resource Use Measurement [6] was searched for a neurological-based questionnaire which was suitable for adaptation for NMOSD. We selected a comprehensive questionnaire originally developed for epilepsy [7, 8], but modified for amyotrophic lateral sclerosis [9] and multiple sclerosis [10]. Additional items were included to account for ophthalmology services. The resource use questionnaire included items on hospital admission (emergency department, outpatient and inpatient visits), primary care services (general practitioner, nurse), tests and investigations, medicines (prescribed, and over-the-counter purchases), personal social services, mobility and any required adaptations, non-medical costs (such as in relation to transport), and indirect costs (based on productivity losses). Patients were asked to provide information on costs which were related and unrelated to NMOSD, in order to ensure that the analysis considered insofar as was possible, those costs which were associated with NMOSD.

An important consideration for self-reported data for resource use was the recall period as this can lead to bias if respondents do not recall some aspects of care when asked. Generally, it is accepted that the longer the recall period the higher the risk of reduced accuracy of the data [11]. As there is no optimal length of recall period, a 3-month recall period was used [12], with the exception of adaptations or any equipment purchased, where a timeframe of the preceding year was given to reflect the infrequency by which patients would receive these high-cost items; and prescribed medicines for which a 1-month recall period was specified.

#### Health outcomes

Health utilities were based on the EQ-5D-5L questionnaire [13], which is a generic, multi-attribute instrument consisting of five dimensions: mobility, self-care, usual activities, pain/discomfort, and anxiety/depression. A total of 3125 possible health states are defined in the EQ-5D-5L, each associated with a corresponding utility score which is anchored at 0 (death) and 1 (perfect health). Negative utility scores indicate states perceived to be worse than death. The EQ-5D-5L value set for England was used, based on a study which followed the EuroQol Group’s international protocol for valuing EQ-5D-5L health states [14]. Subsequent to our study protocol being approved, NICE recommended the use of the EQ-5D-3L mapping function proposed by van Hout et al. [15], and later a mapping function by Hernández Alava et al. [16]. Given also the ongoing research to develop a new UK value set for the EQ-5D-5L [17], we decided to continue with the approach recommended by the EuroQol group, as originally planned. The second part of the EQ-5D-5L consisted of a vertical visual analogue scale (VAS), where 0 represents the worst and 100 represents the best possible health state imaginable. Respondents marked a point on the scale to reflect their overall health on the day of completion.

A recognised limitation of the EQ-5D-5L is that it lacks sensitivity to changes in visual impairment that affects NMOSD patients [18]. The VisQoL was therefore included as a multi-attribute, vision-related utility measure which disaggregates vision into six items [19]. These include: vision related injury, vision and the demands in their life, vision effect on friendship, organising assistance, vision impact on fulfilment of roles and confidence to join everyday activities. The VisQoL value set was derived from a face-to-face time trade-off study which involved 374 participants, with utility anchored at 0 to represent death and 1 representing full health [20]. Missing values in the VisQoL were replaced with the mean of the other items, rounded to the nearest integer [21].

Self-assessed disease severity was assessed using banded scores of the Kurtzke Expanded Disability Status Scale (EDSS) [22], with 0.0 ≤ EDSS ≤ 4.0 representing an ability to walk for at least 500 m without using a stick, splint or other support, or resting; 4.5 ≤ EDSS ≤ 6.5 representing an ability to walk between 20–499 m, using aids such as stick or splint if needed; 7.0 ≤ EDSS ≤ 7.5 corresponding to not being able to walk for more than 5 m, even with aid (such as frame); and 8.0 ≤ EDSS ≤ 9.5 indicating a need for a wheelchair all the time. Patients’ medical records were reviewed by a neurologist from the NMOSD diagnostic and advisory service to ensure that patient-reported scores were in keeping with their recorded disability and visual acuity. Where there were discrepancies, checks were made for data entry errors and confirmation with the patient.

### Informal carers’ questionnaire

Data collection for patients’ informal carers included: (1) their relation to the patient and their caring activities, including the types of activities and the number of hours spent completing these activities (daily or weekly); (2) work and employment, their economic status and income, any days of work missed due to caring activities; and (3) their health-related quality of life and wellbeing.

Carer health utility was measured using the EQ-5D-5L. Carer wellbeing was gauged using the Carer Experience Scale [23], which contains six attributes, including activities, support, assistance, fulfilment, control and relationships, with three levels for each (most, some and few). Attribute level index values enabled the caring experience to be measured and valued through the use of a simple profile measure.

### Recruitment and survey administration

Patients and their carers were recruited via the Walton Centre NHS Foundation Trust, which is one of two specialist centres for NMOSD serving patients from the north of England, Scotland and North Wales. About 200 NMOSD patients are seen by the NMOSD diagnostic and treatment service at the Walton Centre, accounting for approximately a quarter of the total estimated adult NMOSD population in the UK [24].

Patients eligible for enrolment had clinically or laboratory-supported NMOSD diagnosis according to the 2006 criteria of Wingerchuk et al. [25], were at least 18 years of age and spoke English. Informed consent was obtained prior to their participation.

All data were collected via a postal questionnaire, with reminders to complete the forms given at clinic visits. Follow-up questionnaires were scheduled for 6, 9, 12 and 15 months following baseline administration.

### Unit costs

Inpatient and outpatient appointment costs were calculated using gross costing techniques, assuming national averages for nurse support for outpatient procedures in neurology, and consultant-led neurological procedures (Table 1). Ophthalmology appointments related to NMOSD were costed as the weighted mean of face-to-face consultant-led procedures in ophthalmologist and medical ophthalmologist services, and based on the national reference costs [26]. NMOSD inpatient bed-days were costed as a weighted mean of the elective and non-elective admissions for multiple sclerosis patients. The unit costs of appointments with other NHS professionals, such as a psychologist, social worker and physiotherapist, and for personal social services, were obtained from the compendium of Unit Costs of Health and Social Care [27]. The unit costs of medicines were taken from the British National Formulary [3]. Test costs, including computerized tomography scan, ultrasound, X-ray (Direct Access Plain Film), Dual-energy X-ray absorptiometry (DEXA), lumbar puncture (Diagnostic Spinal Puncture—neurology only) were retrieved from the national reference costs [26]. Urine and blood test costs were obtained from the National Clinical Guideline Centre [28]. The costs of adaptations and travel were estimated from patients’ self-reported data. The analysis was based on 2016/17 costs.

**Table 1:** Unit costs

	Unit cost (£)	Reference
*NHS services*		
Emergency department	149.78	26
Admitted to hospital as an inpatient	484.38	26
Inlier bed days	484.37	26
Excess bed days	346.00	26
Doctor hospital outpatient	346.03	27
GP doctor appointment	36.88	27
GP practice nurse	32.40	27
Nurse at home	26.65	27
Nurse hospital	90.81	26
Ophthalmologist hospital	95.22	26
Podiatrist	47.37	27
Specialist Doctor	173.01	26
Specialist nurse	90.81	26
*Tests*		
Urine	3.85	28
Blood	6.00	28
CT	101.57	26
Ultrasound	53.25	26
MRI	144.26	26
X-Ray	29.78	26
DEXA scan	81.15	26
Lumbar puncture	230.77	26

Carer costs—proxy method	Cost (£) per hour	
Personal care /physical care /giving medicines	24.00	27
Dealing with care services /benefits /financial matters	30.00	27
Other practical help	7.90	Minimum wage
Social activities	7.90	Minimum wage

Carer costs—opportunity cost method	Male	Female	
22–29 years	15.34	14.40	29
30–39 years	19.94	18.24	29
40–49 years	22.28	17.62	29
50–59 years	21.62	16.54	29
60+ years	18.60	14.35	29

Two methods were used to estimate the cost of carer activities, the proxy method and the opportunity cost method [29]. For the proxy cost method, informal care costs were matched with those from formal services as follows: personal care, physical help and giving medicines were valued as the time of a formal carer; help dealing with care services or financial matters was assigned a value corresponding to that of a social worker; and other practical help and social activities were estimated at the minimum wage rate (Table 1). The opportunity cost method used the national average hourly wage, stratified by age and sex to estimate the daily cost of caring. To avoid double counting activities that a caregiver may be preforming during the course of the day, a sensitivity analysis was undertaken for the cost of social caring activities. This considered the cost of a hospital sitter (proxy cost), the minimum payment of carers benefit, and the maximum payment of carers benefit (opportunity cost method).

For both carers and patients currently in employment, productivity loss was assessed through the analysis of the rate of sick leave. The productivity of a person was valued at the average market price in terms of age and gende﻿r [30]. For short-term sick leave the labour costs were adjusted to the respondents’ reported missing working hours.

### Statistical analysis

Data from questionnaire responses were analysed descriptively as frequencies, means, standard deviations and ranges. Non-parametric bootstrap analyses (bias-corrected and accelerated) with 10,000 replications were used to estimate the 95% central range (CR) in total costs and utilities, acknowledging the skewness in the distribution of these variables. Data management and statistical analyses were performed using Stata version 13 (StataCorp LP, TX).

## Results

### Patient characteristics

Questionnaire packs were sent to 190 patients, of which 117 (62%) returned at least one completed pack. Fifty-three returned a second questionnaire, 20 a third, 8 a fourth and one patient returned a fifth questionnaire. Participants were predominantly female, with a mean age of 53 years, and had waited 6 years for referral to the specialist NMOSD service (Table 2). The mean length of time since the onset of symptoms was 12 years; and participants reported an average of 3 relapses after their first attack since diagnosis. The majority (56; 50%) of the 111 patients who completed the EDSS questionnaire reported moderate disability (4.5 ≤ EDSS ≤ 6.5).

**Table 2:** Patient demographic and clinical characteristics

Characteristic	Mean (SD, range) or (%)
Total number of patients, N	117
Gender, female N (%)	91 (78%)
Age at baseline, years (SD, range)	53 (15, 18–86)
Age at first onset of symptoms, years (SD, range)	44 (15, 14–85)
Length of time until referral to the Walton centre, years (SD, range)	6 (7, 0–36)
Duration since first attack, years (SD, range)	12 (8, 1–45)
Number of relapses per patient, mean (range)	3 (0–10)
Mild disability (EDSS ≤ 4.0) N (%)	29 (26%)
Moderate disability (4.5 ≤ EDSS ≤ 6.5) N (%)	56 (50%)
Moderate to severe disability (7.0 ≤ EDSS ≤ 7.5) N (%)	14 (13%)
Severe disability (8.0 ≤ EDSS ≤ 9.5) N (%)	12 (11%)

#### Health utilities

Baseline responses to the EQ-5D-5L indicated that 106 (93% of completed questionnaires) patients reported problems in one or more of the dimensions. Thirty-three (29%) reported severe or extreme pain or discomfort, and 14 (12%) were unable to walk (Table 3). For usual activities, 101 (88%) reported difficulty undertaking work, study, housework, family, or leisure activities. Mean utility at baseline was 0.54 (95% CR 0.49, 0.60; n = 113). The mean EQ-5D VAS score was 52.8 (95% CR 48.60, 56.93; n = 113). Longitudinally, EQ-5D-5L utility scores remained consistent with means of 0.56, 0.56 and 0.59 for the second, third and fourth survey.

**Table 3:** Baseline patient responses to the EQ-5D-5L and VisQoL, N(%)

Attributes					
Levels	Mobility	Self-care	Usual Activities	Pain or discomfort	Anxiety or depression
*EQ-5D-5L*					
1	21 (18.2%)	46 (40.4%)	14 (12.2%)	8 (7.0%)	34 (29.6%)
2	22 (19.1%)	26 (22.8%)	32 (27.8%)	29 (25.4%)	46 (40.0%)
3	39 (33.9%)	27 (23.7%)	38 (33.0%)	44 (38.6%)	22 (19.1%)
4	19 (16.5%)	10 (8.8%)	20 (17.4%)	21 (18.4%)	7 (6.1%)
5	14 (12.2%)	5 (4.4%)	11 (9.6%)	12 (10.5%)	6 (5.2%)

Levels	Injury	Demands of Life	Friendships	Assistance	Roles	Confidence
*VisQoL*						
1	48 (49%)	30 (31%)	6 (6%)	40 (41%)	42 (43%)	6 (6%)
2	35 (36%)	18(19%)	77 (79%)	26 (27%)	17 (18%)	48 (49%)
3	10 (10%)	28 (29%)	6 (6%)	8 (8%)	17 (18%)	26 (26%)
4	0 (0%)	11 (11%)	4 (4%)	6 (6%)	9 (9%)	9 (9%)
5	4 (4%)	9 (9%)	2 (2%)	2 (2%)	10 (10%)	6 (6%)
6	–	1 (1%)	1 (1%)	15 (15%)	2 (2%)	2 (2%)
7	–	–	1 (1%)	–	–	–

Ninety-seven (83%) participants completed the VisQoL questionnaire at baseline. Most reported difficulty in one or more dimensions, with the greatest difficulties being in vision making it difficult for people to cope with the demands in their lives, affecting confidence to join in everyday activities, and making it difficult to fulfil the roles they would like to fulfil in life (Table 3). Respondents were least affected by the effect of their vision on the potential for injury or ability to have friendships. The mean VisQoL utility score at baseline was 0.79 (95% CR 0.74, 0.84).

Significant reductions in utility were observed between disease states, ranging from 0.80 for patients who reported EDSS ≤ 4.0, to 0.20 for those with scores 8.0 ≤ EDSS ≤ 9.5 (Table 4). Monotonically decreasing EQ-5D VAS scores and VisQoL utilities were not as apparent with increasing EDSS scores.

**Table 4:** Estimates of patient EQ-5D-5L utilities, EQ-5D VAS and VisQoL utilities, by EDSS scores

EDSS scores (number per banding)	EQ-5D-5L (95% CR, range)	EQ-5D VAS (95% CR, range)	VisQoL (95% CR, range)
EDSS ≤ 4.0 (n = 29)	0.80 (0.75–0.85, 0.44–1.00)	49.41 (43.50–55.32, 10–95)	0.85 (0.77–0.94, 0.23–0.99)
4.5 ≤ EDSS ≤ 6.5 (n = 56)	0.54 (0.48–0.60, − 0.01 to 0.87)	67.37 (59.71–75.03, 30–100)	0.78 (0.70–0.85, 0.1–0.99)
7.0 ≤ EDSS ≤ 7.5 (n = 14)	0.31 (0.12–0.50, − 0.22 to 0.78)	41.79 (30.77–52.80, 10–75)	0.83 (0.71–0.95, 0.37–0.99)
8.0 ≤ EDSS ≤ 9.5 (n = 12)	0.20 (0.02–0.38, − 0.29 to 0.56)	51.81 (39.41–64.23, 25–80)	0.60 (0.34–0.85, 0.23–0.99)
All patients (n = 111)	0.54 (0.49–0.60, − 0.29 to 1.00)	52.77 (48.60–56.93, 10–100)	0.79 (0.74–0.84, 0.11–0.99)

#### Healthcare resource use and costs

Costs were based on responses to baseline questionnaires. Hospitalisation was not common in the patient cohort, with only 10 (9%) of patients reporting that they had been hospitalised in the preceding 3 months. However, patients who had undergone an inpatient stay reported a considerable length of stay, with a mean duration of hospitalisation of 12.5 days (median: 1.5, range: 1–90). Lengths of stay varied by disease severity, ranging from 5 days with EDSS ≤ 4.0, to 90 days with 8.0 ≤ EDSS ≤ 9.5. The mean cost of hospitalisation was £3954 (95% CR £509, £9221).

Table 5 presents the costs by category and EDSS score. Mean total costs increased with disability, from £562 (95% CR £381, £812) in patients with EDSS ≤ 4.0, to £32,717 (95% CR £2888, £98,568) with 8.0 ≤ EDSS ≤ 9.5. Inpatient hospitalisations accounted for the majority of these costs.

**Table 5:** Patient costs over the 3 months preceding the first questionnaire completed – totals and by EDSS score

	Total costs Mean (95% CR)	EDSS ≤ 4.0 Mean (95% CR)	4.5 ≤ EDSS ≤ 6.5 Mean (95% CR)	7.0 ≤ EDSS ≤ 7.5 Mean (95% CR)	8.0 ≤ EDSS ≤ 9.5 Mean (95% CR)
Travel	£69 (£49–£89)	£43 (£14–£84)	£68 (£13–102)	£56 (£3–£110)	£157 (£89–218)
Patient Costs	£704 (£217–£1511)	–	£366 (£33–£1113)	£162 (£2–£324)	£4898 (£1030–£12,984)
GP Practice	£154 (£124–£197)	£93 (£49–£143)	£151 (£110–£199	£225 (£111–£437)	£259 (£153–£419)
Other contacts	£55 (£33–£98)	£12 (£4–£23)	£36 (£19–£67)	£33 (£0–£75)	£269 (£89–539)
Tests	£78 (£61–£104)	£70 (£31–£120)	£78 (£55–£113)	£102 (£29–£189)	£241 (£124–£372)
Medications	£607 (£208–£1459)	£89 (£44–£180)	£1135 (£289–£3422)	£216 (£96–£412)	£408 (£112–£917)
A&E attendances	£70 (£44–122)	£23 (£0–£67)	£70 (£35–£117)	£160 (£0–£366)	£116 (£15–291)
Hospital out-patients	£318 (£245–£420)	£212 (£125–£323)	£322 (£201–£426)	£482 (£270–£957)	£428 (£179–921)
Hospital in-patient stay	£3954 (£509–£9221)	£23 (£0–£90)	£1436 (£22–£3778)	£4670 (£0–£13,829)	£25,951 (£0–£71,746)
Total cost	£5623 (£2096–£12,156)	£562 (£381–£812)	£3674 (£1813–£6347)	£6106 (£923–£20,562)	£32,717 (£2888–£98,568)

*Patient costs* are self-reported by patients, and include private medication, house adjustments; *GP Practice* includes out-of-hours services, practice nurse and GP home visits; *Other contacts* include physiotherapy, occupational health, social work, counselling and psychotherapy

#### Out-of-pocket and productivity losses

Seventeen (15%) patients reported that they had purchased items in the previous year for home adaptations, wheelchairs and mobility scooters, public liability insurance, medication and private prescriptions. The average cost of adaptations was £4843 (95% CR £3273, £6412). Additional travel expenses were reported by 44 (38%) patients, at a mean cost of £80 (95% CR £ 41, £119) over a 3-month period.

Forty-seven patients had left the workforce including 16 due to their long-term illness and retirement. Seven patients stated that their employment situation had been affected due to NMOSD. Only 13 patients responded that they were in paid employment, of which 7 reported taking an average of 30 days off in the previous 3 months because of sickness.

### Carer survey

A total of 123 survey responses was received from 74 informal carers (Table 6). The mean age of carers was 55 (range 22–79), with 75% of carers being 50 years old or more. Most carers were male (61%) and retired (26%), and most were married to the patient (74%) or were the patient’s son or daughter (11%). A higher proportion of male carers (96%) lived with the person they cared for compared to females (72%) and were the spouse/partner of the patient (86%). 55% of female carers cared for their spouse or partner and 30% were looking after other family relatives. Of the carers who responded, only females were caring for non-relatives.

**Tabl﻿e 6:** Carer demographics

Characteristic	Mean (%) (range)
Number	74
Mean age (range)	55 (22–79)
Male (%)	45 (61%)
*Carers age profile (years)*	
20–29	3 (4%)
30–39	5 (7%)
40–49	8 (11%)
50–59	31 (44%)
60–69	17 (24%)
70–79	6 (9%)
*Relationship to the NMOSD patient*	
Spouse/partner (%)	55 (74%)
Son/daughter (%)	8 (11%)
Parent/guardian (%)	5 (7%)
Sibling (%)	2 (3%)
Other non-relative (%)	4 (5%)
*Living arrangements*	
Patient lives with carer	64 (86%)
Patient lives in own home	9 (12%)
Patient lives in Care Home	1 (1%)
*Carer employment status*	
In full time employment	27 (36%)
In part-time employment	8 (11%)
Unemployed and not looking for work	4 (5%)
Unable to work due to caring commitments	15 (20%)
On a government employment or training scheme	1 (1%)
Retired	19 (26%)
*Carer commitments affecting career*	
Yes	25 (34%)
No	46 (62%)
Other	3 (4%)
*Reasons for caring commitments affecting work*	
Lost a paid job and still have not got another one	2 (8%)
Changed the type of job/tasks done	1 (4%)
Lost a paid job but have since got another one	1 (4%)
Changed my place of work	2 (8%)
Changed the number of hours worked	8 (31%)
Unemployed for the last 3 months	2 (8%)
Unemployed then got a paid job	2 (8%)
Opted to take early retirement due to caring commitments	8 (31%)
*Carers’ weekly earnings*	
None	18 (29%)
Less than £99	9 (14%)
£100–£199	9 (14%)
£200–£299	8 (13%)
£300–£399	5 (8%)
£400–£499	8 (13%)
£500–£599	1 (2%)
£600–£699	2 (3%)
£700–£799	2 (3%)
More than £800	1 (2%)

Twenty-five (34%) carers reported being affected by their carer roles (Table 6). Carer-reported EQ-5D-5L utility for baseline responses was 0.85 (95% CR 0.82, 0.89; range 0.3–1.0), and was comparable between males and females. Mean EQ-5D VAS scores were 77 (95% CR 72, 81; range 20–100), and CES index scores were 57.67 (95% CR 52.69, 62.66; range 0–100). The most frequent response to each CES item indicated that most had little support from family, friends, organisations or the government (Table 7). Carers mostly found fulfilment from caring and were able to undertake most desired tasks outside of carer responsibilities.

**Table 7:** Responses to the Carer Experience Scale

Attribute (levels)	N (%)
*Activities outside caring*	
Can do most of the things they want to do	32 (46%)
Can do some of the things they want to do	22 (31%)
Can do a few of the things they want to do	16 (23%)
*Support from family and friends*	
A lot	17 (24%)
Some	23 (33%)
A little	30 (43%)
*Assistance from organisations and the government*	
A lot	3 (5%)
Some	6 (9%)
A little	55 (86%)
*Finding fulfilment from caring*	
Mostly	31 (46%)
Sometimes	27 (40%)
Rarely	9 (13%)
*Level of control over aspects of caring*	
Mostly	28 (41%)
Some	29 (43%)
A few	11 (16%)
*Getting on with the person you care for*	
Mostly	62 (90%)
Sometimes	7 (10%)
Rarely	0 (0%)

#### Carer burden

Of those who responded, 19 (26%) spent between 35 and 49 hours per week caring for patients, spending most of this time on social aspects of caring, physical help and other practical help. Other activities included travel assistance, keeping an eye on patients, help with social activities, physical help, help with administration tasks or financial matters, personal care, and giving medicines.

Twenty-eight (38%) carers reported that their carer commitments affected their employment, although 17 of these did not elaborate on how their employment had changed. Those who reported that they had reduced the number of hours worked, took up new employment, or lost a paying job.

#### Carer costs

The mean daily cost of informal care was estimated to be £144 (95% CR £18, £240) using the proxy good method, and £269 (95% CR £255, £283) using the opportunity cost method (Table 8). With the exception of the costs of social caring activities, the proxy method estimates a higher average cost per task completed.

**Table 8:** Daily costs of informal care

	Time (minutes per day)	Cost (proxy method) Mean (95% CR)	Cost (opportunity cost method) Mean (95% CR)
Personal care	60	£22 (£20.12–£23.51)	£16.36 (£15.04–£17.67)
Physical help	81	£26 (£23.56–£27.25)	£19.09 (£17.78–£20.40)
Helping to deal with care services	23	£6.00 (£5.57–£6.75)	£3.98 (£3.60–£4.35)
Help dealing with paperwork and financial services	36	£13.00 (£12.16–£14.66)	£8.15 (£7.47–£8.83)
Other practical help	82	£12 (£11.47–£13.05)	£30.32 (£28.64–£32.01)
Giving medicines	25	£13 (£11.55–£14.79)	£10.45 (£9.19–£11.69)
Social caring activities	600	£75 (£71–£80)	£189.99 (£179.90–£201.08)
Total	907	£144.25 (£18–£240)	£269.07 (£255.31–£282.85)

## Discussion

### Principal findings

This is the first study to quantify the economic burden of NMOSD on patients and their informal caregivers in the UK. It reveals the high costs of health and social care and private expenditures that are associated with increasing disease severity, as well as the economic impacts on care-giving family members. The mean, total costs of the whole cohort were estimated as £5623 per quarter (equivalent to £22,492 over 1-year), but were higher for patients with 8.0 ≤ EDSS ≤ 9.5, at £32,717 (equivalent to £130,868 over 1-year) mainly due to increased hospitalisation. The association between healthcare costs and EDSS disability scores has been documented previously for patients with multiple sclerosis [31].

Patients with NMOSD report low utility scores on the EQ-5D-5L. Their mean score of 0.54 compares with 0.57 for patients with amyotrophic lateral sclerosis [9] and 0.64 for patients with multiple sclerosis [32]. As the EQ-5D is unresponsive to different levels of visual acuity, our use of the VisQoL aimed to better characterise utilities associated with vision impairment. Our respondents’ mean score of 0.79 is similar to utility scores reported for patients with age-related macular degeneration, diabetic retinopathy or macular oedema [33]. However, a direct comparison of VisQoL and EQ-5D utilities is not possible given their different constructs.

Carer-reported EQ-5D-5L utility was 0.85 which is higher than reported for carers for people with dementia (0.78), but carers for NMOSD are younger by around a decade [34]. However, the burden on carers is significant, with over 22% of carers spending more than 100 hours per week caring for NMOSD patients, and 40% reporting impact on their employment. On average, patients were provided about 15 hours per day each day of the year, which we estimate costs between £144 and £269 per day, depending on the method of analysis. This corresponds to between £13,150 and £24,560 over 3-months (or £52,600 to £98,240 over 1-year).

### Comparison with other research

A previous study conducted in a small sample of 21 patients with NMOSD in the USA and which utilised the EQ-5D-5L, yielded higher utility of 0.74 [35], but this analysis applied the EQ-5D-3L crosswalk [15] making the values incomparable. A cost study based on US claims database, found that patients with highly active NMOSD had approximately a 10-times higher hospital inpatient admission rate compared with patients without NMOSD [36]. Annual mean costs of inpatient hospitalisation for NMOSD patients was US$29,054 (approximately £22,800 at 2019 prices), which compares to £15,816 in the present analysis. A further US study estimated the mean, annualised all-cause healthcare expenditure among patients with NMOSD was $60,599 (approximately £45,400) [37]. However, making comparisons across health systems, has little validity given the significant differences in prices, pathways of care and how healthcare is financed.

### Strengths and limitations

Our study has strengths in having recruited a significant proportion of UK patients with NMOSD. The findings are therefore likely to be generalisable to the whole of the UK. Examining informal carer costs and health impacts adds value to the analysis given the significance of the spillover effects in the context of chronic neurological diseases such as NMOSD.

There are some limitations with this study. Firstly, the questionnaire was for self-completion and this reliance on patients can lead to problems including recall and social desirability bias. Patients who may be more engaged with the service, and carers who are less burdened may be more likely to report, although we have no evidence for this. Secondly, completion rates of follow-up questionnaires was low, meaning that a robust longitudinal analysis was not possible. Costs and health-related quality of life are likely to change over time, particularly during episodes of relapses. In relation to costs, we focused on resources that patients reported to be related explicitly to NMOSD. While this approach has the advantage of being conservative, it also represents a lower bound, as costs of NMOSD are amplified by comorbidities [38]. Also, indirect costs were limited to productivity losses; other costs, such as due to premature mortality or retirement were not collected. With regards to outcomes, the study utilised the 2006 criteria for NMOSD as it was well validated, although broader criteria were introduced in 2015 [39]. Patients were also asked to self-assess their level of disability based on bandings of EDSS scores, presented in terms of their ability to walk. The EDSS measure is limited by not being disease specific nor does it include any reference to optic neuritis or other disabilities that affect patients with NMOSD [40]. Finally, the VisQol has limited generalisability in that the value set is based on mapping onto AQoL-7D utilities, which are in turn derived from Australian patients with impaired vision. Alternative instruments such as the bolt-on vision dimension for the EQ-5D may have been more appropriate [41].

## Conclusions

This research represents a significant contribution to documenting and quantifying the resource use, costs and health outcomes of patients with NMOSD in the UK. The study also shows the substantial amount of informal care provided by family members and impacts on their health. The inclusion of carer health-related quality of life in economic evaluations is relatively uncommon but has implications for calculating the cost-effectiveness of treatments. NICE specifies that economic evaluations should include direct health effects for carers where relevant. A recent review of technology appraisals [42] highlighted the significant impact of the inclusion of carer EQ-5D utility scores on estimates of the incremental cost-effectiveness ratios. Economic evaluations of treatments for NMOSD that consider the broader implications of treatments on carer wellbeing and costs are more likely to demonstrate cost-effectiveness.

The study findings have value for decision-makers who may want to highlight the burden of a disease beyond measures of disease incidence, prevalence, morbidity and mortality. The data are also compatible for future health economic analyses of interventions for NMOSD, as they report health state costs and utilities relevant to UK populations.

## Data Availability

Study participants did not consent explicitly for their responses to be shared publicly.
